# A clinically-relevant mouse model that displays hemorrhage exacerbates tourniquet-induced acute kidney injury

**DOI:** 10.3389/fphys.2023.1240352

**Published:** 2023-11-08

**Authors:** Balamurugan Packialakshmi, David M. Burmeister, Joseph A. Anderson, Judah Morgan, Georgetta Cannon, Juliann G. Kiang, Yuanyi Feng, Sang Lee, Ian J. Stewart, Xiaoming Zhou

**Affiliations:** ^1^ Department of Medicine, Uniformed Services University of the Health Sciences, Bethesda, MD, United States; ^2^ Department of Laboratory Animal Resources, Uniformed Services University of the Health Sciences, Bethesda, MD, United States; ^3^ Internal Medicine Residency Program at Madigan Army Medical Center, Joint Base Lewis-McChord, Tacoma, WA, United States; ^4^ Armed Forces Radiobiology Research Institute, Uniformed Services University of the Health Sciences, Bethesda, MD, United States; ^5^ Department of Pharmacology and Molecular Therapeutics, Uniformed Services University of the Health Sciences, Bethesda, MD, United States; ^6^ Department of Biochemistry and Molecular Biology, Uniformed Services University of the Health Sciences, Bethesda, MD, United States

**Keywords:** lower limb, ischemia/reperfusion, rhabdomyolysis, systemic inflammation, lung injury, liver injury

## Abstract

Hemorrhage is a leading cause of death in trauma. Tourniquets are effective at controlling extremity hemorrhage and have saved lives. However, tourniquets can cause ischemia reperfusion injury of limbs, leading to systemic inflammation and other adverse effects, which results in secondary damage to the kidney, lung, and liver. A clinically relevant animal model is critical to understanding the pathophysiology of this process and developing therapeutic interventions. Despite the importance of animal models, tourniquet-induced lower limb ischemia/reperfusion (TILLIR) models to date lack a hemorrhage component. We sought to develop a new TILLIR model that included hemorrhage and analyze the subsequent impact on kidney, lung and liver injuries. Four groups of mice were examined: group 1) control, group 2) hemorrhage, group 3) tourniquet application, and group 4) hemorrhage and tourniquet application. The hemorrhagic injury consisted of the removal of 15% of blood volume through the submandibular vein. The tourniquet injury consisted of orthodontic rubber bands applied to the inguinal area bilaterally for 80 min. Mice were then placed in metabolic cages individually for 22 h to collect urine. Hemorrhage alone did not significantly affect transcutaneous glomerular filtration rate (tGFR), blood urea nitrogen (BUN) or urinary kidney injury molecule-1 (KIM-1) levels. Without hemorrhage, TILLIR decreased tGFR by 46%, increased BUN by 162%, and increased KIM-1 by 27% (*p* < 0.05 for all). With hemorrhage, TILLIR decreased the tGFR by 72%, increased BUN by 395%, and increased urinary KIM-1 by 37% (*p* < 0.05 for all). These differences were statistically significant (*p* < 0.05). While hemorrhage had no significant effect on TILLIR-induced renal tubular degeneration and necrosis, it significantly increased TILLIR-induced lung total injury scores and congestion, and fatty liver. In conclusion, hemorrhage exacerbates TILLIR-induced acute kidney injury and structural damage in the lung and liver.

## Introduction

Hemorrhage accounts for approximately 40% of deaths in traumatic injury worldwide (Curry et al., 2011). Tourniquets are the first-line therapy to control extremity hemorrhage both in civilian and military care settings (Kragh et al., 2008; Scerbo et al., 2017; Muñoz et al., 2020; Gallo et al., 2021; Hinojosa-Laborde et al., 2022). It is estimated that tourniquets may have saved as many as 2,000 lives in the U.S. Military operations in Iraq and Afghanistan (Andersen et al., 2012). Tourniquets are also used as an adjunct procedure in limb surgery to create a bloodless field (Gallo et al., 2021; Tirumala et al., 2021). However, prolonged application and release of tourniquets induces ischemia/reperfusion injury to limbs, releasing myoglobin, damage-associated molecular patterns, cytokines, chemokines, and other harmful molecules, leading to systemic inflammation (Yassin et al., 1998; Cai et al., 2009; Foster et al., 2017). Systemic inflammation increases vascular permeability and leakage, resulting in hypovolemia and hypoperfusion of the kidney and other organs (De Rosa et al., 2018; Simon et al., 2018). Therefore, prolonged use of tourniquets can induce secondary distal organ injuries such as acute kidney, lung and liver injures (Morsey et al., 2003; Arora et al., 2015; Chavez et al., 2016; De Rosa et al., 2018; Leurcharusmee et al., 2018; Kasepalu et al., 2020; BenÍtez et al., 2021). The incidences of acute kidney injury (AKI) associated with tourniquet use range from 0.8% to 17.2%, depending on whether patients are diabetic (Morsey et al., 2003; Arora et al., 2015; Chavez et al., 2016; De Rosa et al., 2018; Leurcharusmee et al., 2018; Kasepalu et al., 2020; BenÍtez et al., 2021; Pottecher et al., 2021; Paquette et al., 2022). There is no specific prevention or treatment for tourniquet-induced AKI or other organ injury (Zhou, 2023).

An animal model closely mimicking the clinical setting is important to understanding pathophysiology and testing therapeutic approaches. Although pigs are more similar to humans in physical size, physiology and immunology than rodents (Packialakshmi et al., 2020; Zhou, 2022), rodents are easy to handle, quickly reproduce and have contributed significantly to deciphering pathophysiology and developing novel therapies. As such, a majority of tourniquet-induced lower limb ischemia/reperfusion (TILLIR) injury in the literature has been produced in rodents, and a variety of therapies have been tested using rodent models (Klausner et al., 1989; Adachi et al., 2006; Hsu et al., 2012; Mansour et al., 2014; Kao et al., 2015a; Kao et al., 2015b; Karahan et al., 2016; Onody et al., 2016; Foster et al., 2017; Shih et al., 2018; İnce et al., 2019).

However, these models were produced by applying a tourniquet either unilaterally or bilaterally to the hindlimbs without inducing hemorrhage. In a clinical setting, however, it is unlikely that a tourniquet can be placed prior to significant blood loss. Hemorrhage induces hypoperfusion, metabolic derangement, coagulopathy and systemic inflammation, all of which play a critical role in the pathogenesis of multi-organ injury (Denk et al., 2018). As a result, these models have missed a crucial factor in TILLIR-induced organ injury. To address this gap, we sought to develop a novel, clinically relevant mouse model of TILLIR by inducing hemorrhage prior to tourniquet application and examined the subsequent impact of TILLIR, with and without hemorrhage, on end-organ damage with a focus on AKI. We hypothesized that hemorrhage would exacerbate TILLIR-induced organ injury.

## Materials and methods

### Mice

**FIGURE 1:**
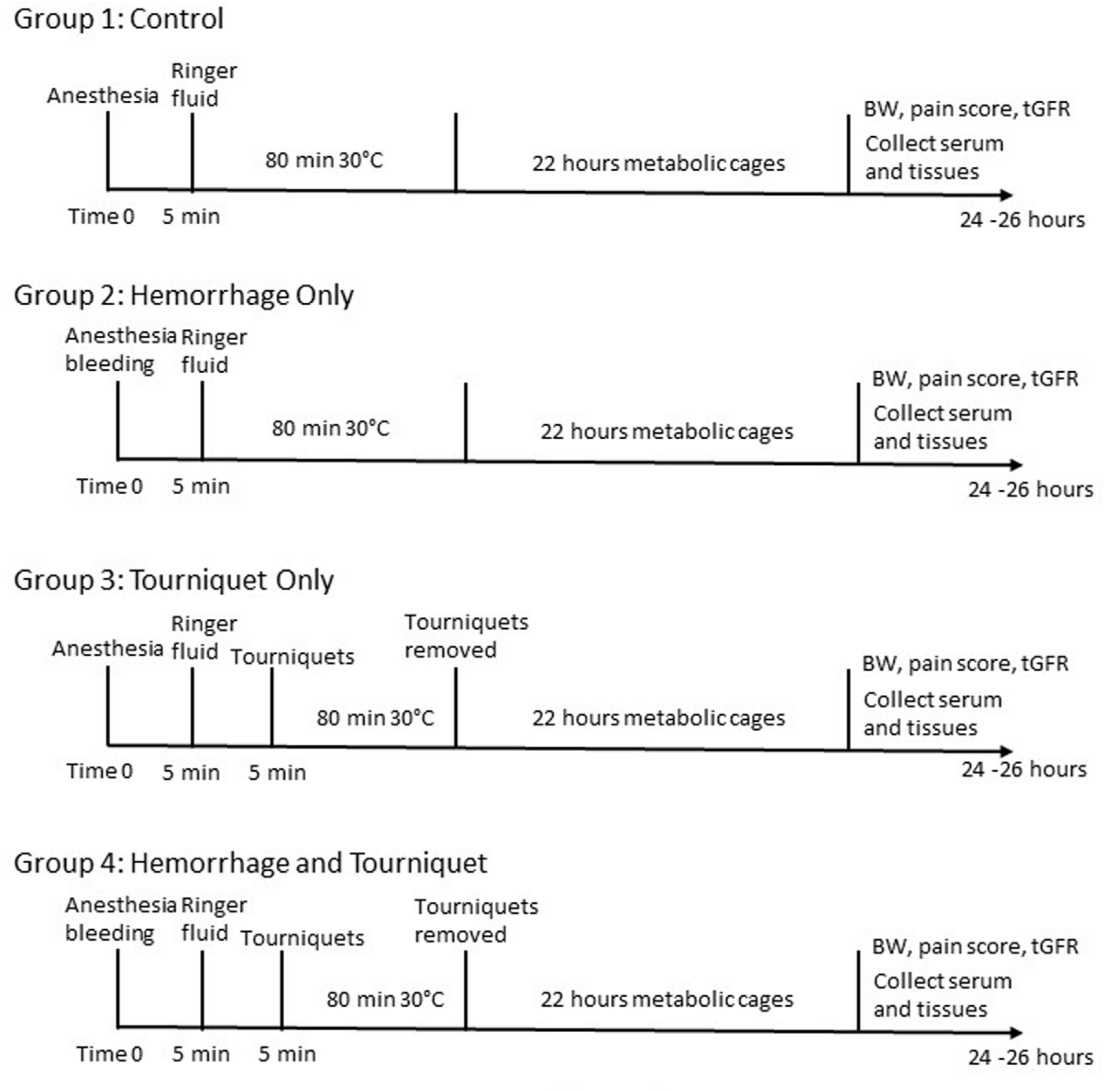
Experimental design and procedures. BW, body weight; tGFR, transcutaneous glomerular filtration rate.

The protocol for use of mice was approved by the institutional animal care and use committee of the Uniformed Services University of the Health Sciences. Male C57BL/6 mice (8–10 weeks old, The Jackson Laboratory) were chosen because they are more prone to develop AKI compared to females (Park et al., 2004; Kher et al., 2005). Mice were kept on a 12:12 light:dark cycle with regular food and water *ad libitum*. Mice were adapted to the facility for at least 3 days before their use for experiments. The experimental design and procedures are outlined in Figure 1. Mice were divided into 4 groups. Group 1 is the control in which mice received the same procedures as other groups, but did not undergo hemorrhage or tourniquet application. Group 2 mice were bled up to 15% blood volume (Class I shock) through the submandibular vein. Group 3 mice had tourniquets (an orthodontic rubber band, 3.2 mm, heavy force 4.5 Oz, purchased from Amazon) applied bilaterally in the inguinal regions for 80 min. Group 4 mice received both tourniquets (80 min) and hemorrhage (15% of blood volume). Blood volume was calculated using an estimation of 1.4 mL/20 g of body weight. Mice were weighed and injected intraperitoneally with ketamine (100 mg/kg) and xylazine (4 mg/kg) in sterile PBS for anesthesia. Buprenex (0.1 mg/kg) was injected subcutaneously for analgesia. All mice were resuscitated with 1 mL Ringer’s solution subcutaneously with 0.5 mL on each side. Mice were shaved in the right lumbar region to prepare for measuring transcutaneous glomerular filtration rate (tGFR) the next day. All mice were placed at 30°C in an incubator while they were under anesthesia to maintain body temperature. All mice were then placed individually in metabolic cages to collect urine for 22 h. At the end of 22 h, mice were again weighed and scored on the severity of the symptoms such as hind limb mobility, appearance and provoked response (Koch et al., 2016). After tGFR measurements, the mice were euthanized using an intraperitoneal injection of ketamine (100 mg/kg) and xylazine (4 mg/kg), followed by cervical dislocation.

### tGFR measurement

tGFR was measured transdermally with the clearance of FITC-sinistrin (Scarfe et al., 2018). Briefly, under isoflurane anesthesia, mice were injected with FITC-sinistrin (5 mg/100 g body weight; Mannheim Pharma and Diagnostics GmbH) via retro- orbital route and the disappearance of the FITC fluorescence was monitored by a Medibeacon NIC-Kidney units with internal memory (Mannheim Pharma and Diagnostics GmbH) mounted on the lumbar region. The animals were placed in cages individually for an hour and then the data were transferred to a computer and analyzed with the MPD 1.0 software. The half-time of reduction in the blood FITC-sinistrin concentrations is proportional to GFR, which was calculated based on the manufacturer’s formula.

### Blood urea nitrogen measurement

Blood urea nitrogen (BUN) levels were detected with the diacetyl monoxime method (Rahmatullah and Boyde, 1980). Briefly, a chromogen was freshly prepared by mixing Reagent A (10 mg of ferric chloride dissolved in 10 mL of 1.48 M phosphoric acid mixed with 30 mL of 5.52 M sulfuric acid and 60 mL of distilled water) and Reagent B (50 mg of diacetyl monoxime and 1 mg of thiosmicarbazide dissolved in 10 mL of water) at 2:1. The serum samples were diluted 10 times with de-ionized water. Proteins in the diluted serum were precipitated with 0.61 M trichloroacetic acid solution, and the supernatants were incubated with the chromogen at 80°C for 5 min. The absorption was measured at 490 nm.

### Urinary KIM-1 assay

Urinary kidney injury molecule (KIM-1) was measured with the Mouse KIM-1 ELISA kit from Abcam (ab213477) according to the manufacturer’s protocol and normalized to urinary creatinine levels that were measured with the Jaffe’s method (Toora and Rajagopal, 2002). Briefly, 50 μL mouse urine sample (diluted 5 times) was incubated with 25 µL mixture of 0.75 N NaOH and 1% picric acid (1:1) at room temperature for 15 min. The absorption was recorded at 490 nm.

### Histology

The kidney, lung, and liver fixed with 10% formalin for 48 h and then transferred to 70% ethanol. The tissues were embedded in a Paraplast Plus Tissue Infiltration/Embedding Medium (McCormick Scientific), sectioned at 4 μm, and stained with hematoxylin/eosin. Two to four sections of each tissue were examined by a board-certified veterinary pathologist in a blinded fashion using an Olympus BX43 light microscope with magnification ranging from 1.25X-60X. The kidney histopathology was scored based on tubular degeneration and necrosis, cast, congestion and hemorrhage. Lung histopathology was graded based on hemorrhage, congestion, pigment, edema, atelectasis and bronchio-alveolar hyperplasia. Liver histopathology was quantified based on fatty change, infiltration of neutrophils and mononuclear cells, oval cell hyperplasia, focal necrosis, congestion, karyocytomegaly, and multinucleated hepatocytes. The total score is the summation of all averages of histological change in an organ. Tissues were scored semiquantitatively by determining the percentage of relevant cells affected as follows: 0 = Normal: tissue considered to be normal under the conditions of the study. 1 = Minimal: amount of change barely exceeds normal, up to 5%; 2 = Mild: the lesion is easily identified but of limited severity, 6%–15%; 3 = Moderate: the lesion is prominent, but there is significant potential for increased severity, 16%–50%; 4 = Marked: the lesion occupies the majority of the examined tissue section but there is still potential for increased severity, 51%–90%; 5 = Severe: the degree of change is as complete as possible and unlikely to be potential for increased severity, >90%.

### Immunohistochemistry

Immunohistochemical staining of KIM-1 was performed essentially as previously described (Packialakshmi et al., 2022). Briefly, paraffin-embedded kidney slides (4 μm) were deparaffinized and rehydrated sequentially with xylene, ethanol, and water. Endogenous peroxidase was blocked using a blocking buffer from a DAB (3,3′-Diaminobenzidine) kit (AB64264, Abcam), following the manufacturer’s protocol. Antigens were exposed by incubating the slides in Antigen Retrieval Buffer (BUF025A, Bio-Rad) at 90°C for 20 min. Non-specific binding was inhibited using a blocking solution included in the DAB kit. Subsequently, slides were incubated with an anti-KIM-1 antibody (1:500 dilution, PA520244, Thermo Fisher) at room temperature for 60 min. Following washing, KIM-1 antibody binding was visualized using the DAB kit and quantified with ImageJ. The percentage of pixels in the cortex above a set threshold represented the protein abundance of KIM-1. Nuclei were stained with 1% Methyl Green (4800-30-18, R&D Systems).

### Serum cytokine and chemokine assay

Blood was collected via cardiac puncture using a heparin-free needle and syringe, and then transferred into an Eppendorf tube. The sample was allowed to sit at room temperature for 30 min before being centrifuged at 1,400 rpm for 15 min at 4°C. Serum was then collected. A total of 23 cytokine and chemokine concentrations in serum were analyzed using the Bio-Plex Pro^™^ Mouse Cytokine Panel Plex (Bio-Rad) as previously described (Kiang et al., 2017). These cytokines and chemokines were IL-1α, IL-1β, IL-2, IL-3, IL-4, IL-5, IL-6, IL-9, IL-10, IL-12 (p40), IL-12 (p70), IL-13, IL-17, eotaxin, G-CSF, GM-CSF, IFN-γ, KC, MCP-1, MIP-1α, MIP-1β, RANTES, and TNF-α. Briefly, serum from each mouse was diluted four-fold and examined. The cytokine and chemokine levels were analyzed using Bio-Plex 200 (Bio-Rad) and quantified using MiraiBio MasterPlexH CT and QT Software (Hitachi Software Engineering America Ltd.), and concentrations were expressed in pg/mL.

### Statistical analysis

Data are expressed as means ± standard error of the mean. Data were analyzed with two-way ANOVA (GraphPad Prism 9.0.2). After identifying the overall significant effects of tourniquet and hemorrhage, multiple comparisons were made with Tukey’s analysis. The significant effects between tourniquet and control and tourniquet and tourniquet plus hemorrhage are reported. *p* < 0.05 was considered significant.

## Results

### Hemorrhage worsens TILLIR-induced reduction in the renal function

**FIGURE 2:**
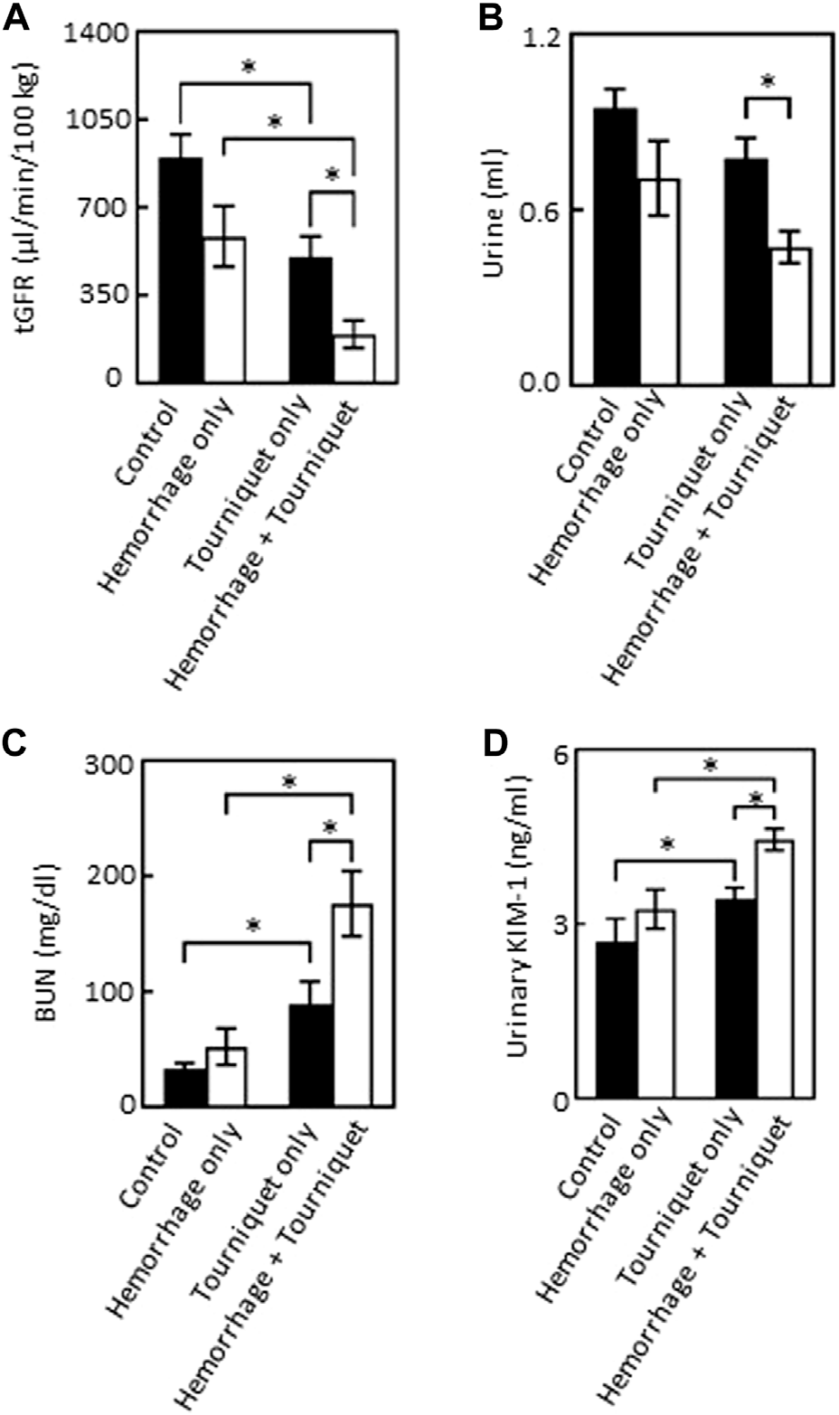
Hemorrhage exacerbates TILLIR-induced reduction in the renal function as assessed by tGFR, urinary output, BUN and urinary KIM-1 levels. **(A)** Tourniquet alone reduced tGFR as compared with the control, addition of hemorrhage further decreased tourniquet-induced reduction of tGFR compared either with hemorrhage only or tourniquet only. The tGFR was measured by disappearance of FITC-sinistrin from the blood. *n* = 5 for the control, *n* = 6 for the hemorrhage only, *n* = 12 for the tourniquet only, and *n* = 11 for the hemorrhage + tourniquet. **(B)** Tourniquet alone did not significantly reduce urinary output, but combining tourniquet with hemorrhage decreased urinary output as compared with the tourniquet only group. Urine was collected through metabolic cages for 22 h *n* = 5 for the control, *n* = 6 for the hemorrhage only, *n* = 11 for the tourniquet only or hemorrhage + tourniquet. **(C)** Tourniquet alone increased serum urea nitrogen (BUN) as compared with the control, the combination of tourniquet with hemorrhage further elevated tourniquet-induced increase in BUN compared either with hemorrhage only or tourniquet only. BUN was measured with a colorimetric method. *n* = 5 for the control or hemorrhage only, *n* = 9 for the tourniquet only, and *n* = 10 for hemorrhage + tourniquet. **(D)** Tourniquets alone increased urinary KIM-1 level as compared with the control. Adding hemorrhage further elevated tourniquet-induced increase in urinary KIM-1 concentrations compared either with hemorrhage only or tourniquet only. The KIM-1 concentrations were measured with an ELISA kit from Abcam (ab213477). *n* = 5 for the control or hemorrhage only, *n* = 10 for the tourniquet only and hemorrhage + tourniquet, respectively. *p < 0.05, Two-way ANOVA.

We found that hemorrhage alone had no significant effect on tGFR, urine output, BUN or urinary KIM-1 levels as compared with the control group. Tourniquet alone significantly reduced tGFR from 928 ± 215 μL/min/100 g to 502 ± 87 μL/min/100 g, and increased BUN from 30.5 ± 7.8 mg/dL to 80.0 ± 22.2 mg/dL, urinary KIM-1 from 2.7 ± 0.4 ng/mL to 3.4 ± 0.2 ng/mL and pain score from zero to 2.5 without significantly affecting urinary output (0.9 ± 0.2 mL vs. 0.8 ± 0.1 mL) compared with the control group. However, the combination of hemorrhage with tourniquets significantly reduced tGFR and urinary output by 61% and 38%, and increased BUN, KIM-1 and pain score by 127%, 24%, and 35%, respectively, compared with tourniquet alone (Figure 2; Table 1). Neither hemorrhage alone nor the combination of hemorrhage with tourniquets had a significant effect on body weight (Table 1).

**TABLE 1:** Effects of tourniquets and hemorrhage on body weight.

	Pain score	Body weight (g)		Difference	n
		At beginning	At end		
Control	0	23.8 ± 1.4	21.6 ± 1.5	2.2 ± 0.4	5
Hemorrhage only	0.2 ± 0.2	22.7 ± 0.9	20.6 ± 0.8	2.1 ± 0.5	6
Tourniquet only	2.6 ± 0.2	22.8 ± 0.4	21.6 ± 0.4	1.2 ± 0.2	12
Hemorrhage + Tourniquet	3.5 ± 0.3*	22.7 ± 0.6	21.4 ± 0.5	1.4 ± 0.4	11

^*^ *p* < 0.05 vs. Tourniquet only.

### Hemorrhage potentiates TILLIR-induced structural damages in the kidney, lung and liver

**FIGURE 3:**
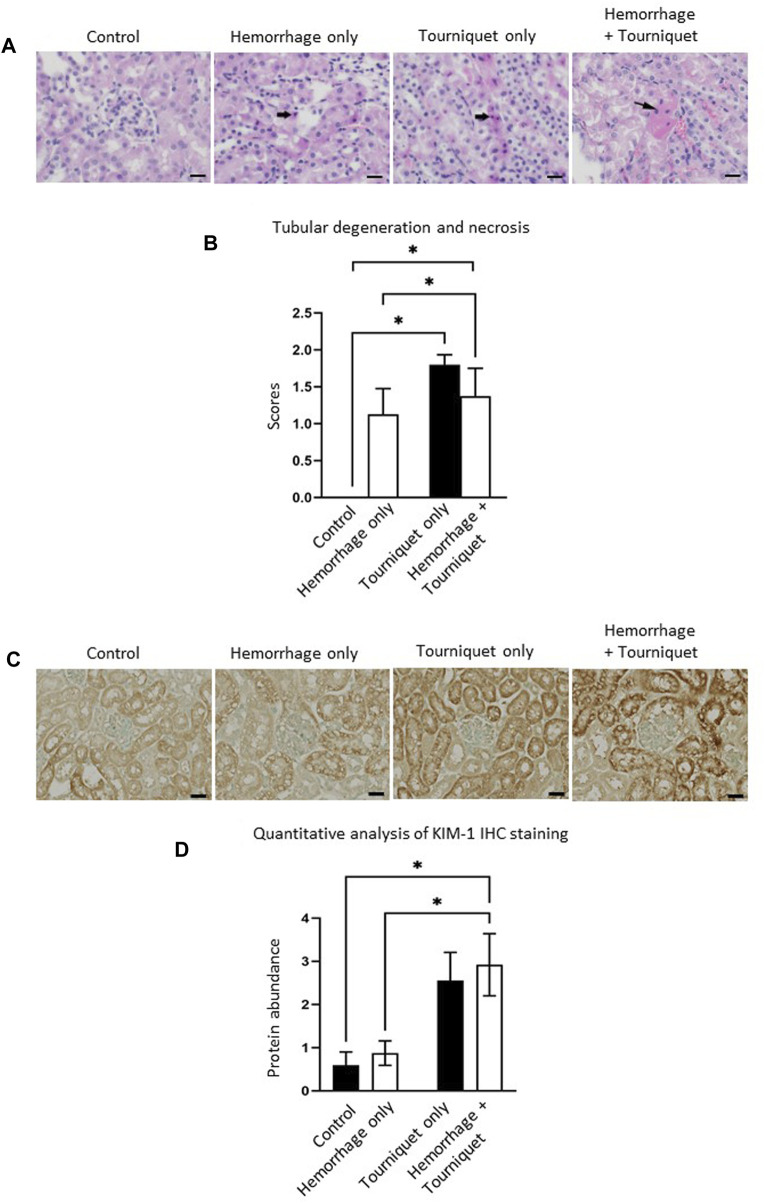
Hemorrhage potentiates TILLIR-induced injury in the renal cortex. **(A)** Representatives of TILLIR-induced renal tubular degeneration and necrosis. Increased hematoxylin/eosin staining indicates tubular degeneration and necrosis (arrow). **(B)** Compared with Control, TILLIR increased renal tubular degeneration and necrosis, but hemorrhage had no additive effect. **(C)** Representatives of TILLIR-induced increase of KIM-1 immunohistochemical staining. **(D)** TILLIR did not significantly increase KIM-1 protein abundance in the absence of hemorrhage. However, it showed a significant increase in KIM-1 protein abundance in the presence of hemorrhage when compared to the hemorrhage only group. In A and B, *n* = 6 for the control, *n* = 8 for the hemorrhage only, *n* = 9 for the tourniquet only and hemorrhage + tourniquet, respectively. In **(C,D)**, *n* = 8. *p < 0.05, Two-way ANOVA. Scale bars: 20 µm.

**FIGURE 4:**
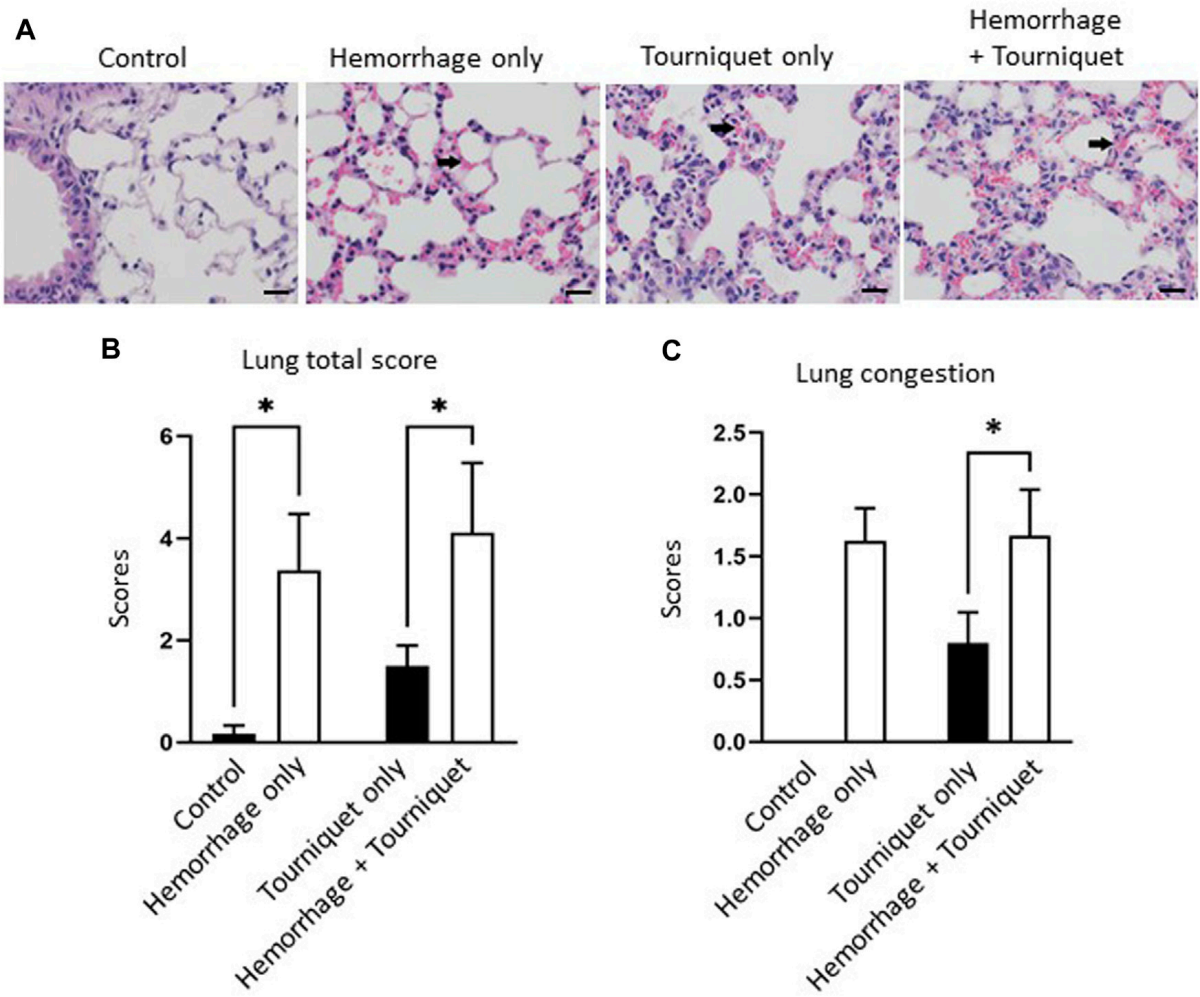
Hemorrhage potentiates TILLIR-induced structural damage in the lung. **(A)** Representatives of TILLIR-induced congestion in the lung (arrow). **(B)** Hemorrhage only increased total lung injury scores compared with the control, whereas tourniquet only did not. However, addition of hemorrhage increased total lung injury scores compared with tourniquet only. **(C)** Neither hemorrhage only nor tourniquet only induced congestion, but combination of these two did compared with tourniquet only. *n* = 6 for the control, *n* = 8 for the hemorrhage only, *n* = 9 for the tourniquet only and hemorrhage + tourniquet, respectively. *p < 0.05, Two-way ANOVA. Scale bars: 20 µm.

**FIGURE 5:**
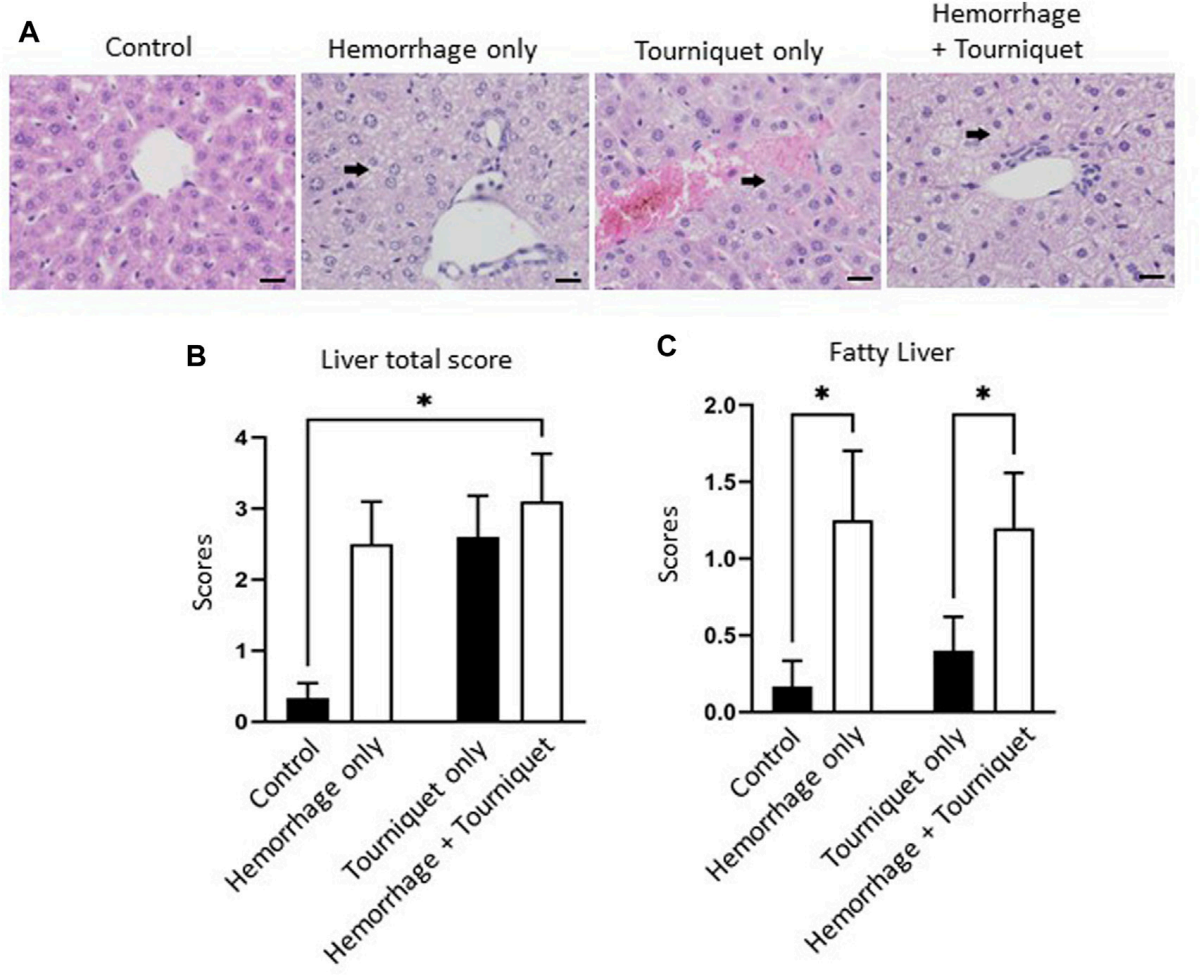
Hemorrhage potentiates TILLIR-induced structural damage in the liver. **(A)** Representatives of TILLIR-induced fat deposit in the liver (arrow). **(B)** Compared with the control, neither hemorrhage only nor tourniquet only significantly increased total liver injury scores, but combination of the two did. **(C)** In the absence of hemorrhage, tourniquet only did not increase fatty liver compared with the control, although hemorrhage only did. However, hemorrhage potentiated tourniquet-induced fatty liver. *n* = 6 for the control, *n* = 8 for the hemorrhage only, *n* = 9 for the tourniquet only and hemorrhage + tourniquet, respectively. *p < 0.05, Two-way ANOVA. Scale bars: 20 µm.

Hemorrhage only, tourniquet only or hemorrhage combined with tourniquet had no significant effect on total injury scores in the kidney compared with the control or among themselves (data not shown). TILLIR caused renal tubular degeneration and necrosis irrespective of hemorrhage, and combination of hemorrhage and tourniquet had no additive effect (Figure 3). In eight experiments, TILLIR exhibited a tendency to elevate the abundance of KIM-1 protein in the renal cortex when compared to the control group in the absence of hemorrhage. However, TILLIR showed a significant increase in KIM-1 protein abundance in the presence of hemorrhage when compared to the hemorrhage only (Figure 3). Compared with the control, hemorrhage only significantly increased total lung injury scores, whereas tourniquet only did not. However, combination of hemorrhage with tourniquet increased total lung injury scores as well as congestion compared with tourniquet only (Figure 4). Compared with the control, neither hemorrhage only nor tourniquet only significantly increased total liver injury scores, but combination of the two did (Figure 5). In the absence of hemorrhage, tourniquet only did not significantly increase fatty liver compared with the control, although hemorrhage only did. However, hemorrhage potentiated tourniquet-induced fatty liver (Figure 5). Averages of histopathological scores in their respective categories were provided in Table 2.

**TABLE 2:** Averages of histopathological scores in their respective categories.

Kidney	Control	Hemorrhage only	Tourniquet only	Hemorrhage + Tourniquet
Tubular degeneration and necrosis	0.00 ± 0.00	1.13 ± 0.35	1.80 ± 0.13*	1.38 ± 0.38^#^
Cast	0.00 ± 0.00	0.13 ± 0.13	0.00 ± 0.00	0.25 ± 0.16
Congestion	0.00 ± 0.00	0.88 ± 0.30	1.05 ± 0.33	1.00 ± 0.38
Hemorrhage	0.00 ± 0.00	0.25 ± 0.16	0.00 ± 0.00	0.13 ± 0.13
Total scores	0.00 ± 0.00	2.38 ± 0.78	2.80 ± 0.39	2.75 ± 0.88
Lung				
Congestion	0.00 ± 0.00	1.63 ± 0.26	0.80 ± 0.25	1.67 ± 0.37^&^
Hemorrhage	0.17 ± 0.17	0.88 ± 0.44	0.50 ± 0.22	0.89 ± 0.45
Pigment	0.00 ± 0.00	0.38 ± 0.26	0.10 ± 0.10	0.44 ± 0.29
Edema	0.00 ± 0.00	0.25 ± 0.25	0.00 ± 0.00	0.22 ± 0.22
Atalectasis	0.00 ± 0.00	0.25 ± 0.25	0.00 ± 0.00	0.67 ± 0.33
Bronchio-Alveolar hyperplasia	0.00 ± 0.00	0.00 ± 0.00	0.10 ± 0.10	0.00 ± 0.00
Total scores	0.17 ± 0.17	3.38 ± 1.10*	1.50 ± 0.40	4.11 ± 1.37^&^
Liver				
Fatty change	0.17 ± 0.17	1.25 ± 0.45*	0.40 ± 0.22	1.20 ± 0.36^&^
Neutrophil infiltration	0.17 ± 0.17	0.00 ± 0.00	0.10 ± 0.10	0.20 ± 0.20
Mononuclear cell infiltration	0.00 ± 0.00	0.13 ± 0.13	0.10 ± 0.10	0.00 ± 0.00
Oval cell hyperplasia	0.00 ± 0.00	0.00 ± 0.00	0.30 ± 0.15	0.40 ± 0.16
Focal necrosis	0.00 ± 0.00	0.00 ± 0.00	0.10 ± 0.10	0.20 ± 0.20
Congestion	0.00 ± 0.00	0.63 ± 0.32	0.90 ± 0.31	1.10 ± 0.31
Karyocytomegaly	0.50 ± 0.34	1.00 ± 0.38	0.80 ± 0.25	1.40 ± 0.34
Multinucleated hepatocytes	0.83 ± 0.31	1.00 ± 0.38	0.70 ± 0.26	1.30 ± 0.30
Total scores	1.67 ± 0.61	4.00 ± 0.98	3.40 ± 0.50	5.80 ± 1.28*

^*^p < 0.05 vs. Control. ^#^p < 0.05 vs. Hemorrhage only. ^&^p < 0.05 vs. Tourniquet only.

### Hemorrhage only has a minor effect on TILLIR-induced cytokine and chemokine profiles in the serum

Tourniquets alone increased the concentrations of various cytokines and chemokines in the serum as compared with the control group (Table 2). Addition of hemorrhage had no significant effect on a majority of tourniquet alone-induced increases of cytokines or chemokines except for decreasing IFN-γ and RANTES levels in the serum (Table 3).

**TABLE 3:** Effects of hemorrhage on serum cytokine and chemokine concentrations (pg/mL).

	Control	Hemorrhage only	Tourniquet only	Hemorrhage + Tourniquet
IL-1α	4.2 ± 3.7	1.9 ± 1.7	27.3 ± 8.0	15.6 ± 5.8
IL-1β	7.0 ± 4.2	10.1 ± 3.3	25.1 ± 5.2	14.8 ± 3.1
IL-5	11.7 ± 10.5	69.6 ± 42.4	208.4 ± 61.9	402.2 ± 103.7*
IL-6	261.4 ± 168.8	335.5 ± 216.4	1637.4 ± 370.3*	803.9 ± 195.1
IL-10	310.9 ± 278.0	461.4 ± 412.7	3558.7 ± 965.2	2652.6 ± 926.0
IL-12 (p40)	2588.2 ± 982.6	2678.8 ± 637.2	3514.1 ± 1081.3	1245.3 ± 199.8
IL-17	ND	247.7 ± 172.3	604.0 ± 221.4	147.5 ± 71.0
KC	2149.5 ± 596.9	1776.3 ± 344.5	4594.8 ± 1290.5	2840.0 ± 354.2
G-CSF	5026.1 ± 2434.5	9214.1 ± 2502.9	28939.3 ± 6221.4*	17989.6 ± 3376.9
GM-CSF	59.8 ± 53.5	225.9 ± 116.1	821.7 ± 152.2	532.9 ± 123.7
Eotaxin	15271.5 ± 1372.3	23218.4 ± 4129.9	37404.5 ± 6012.5	29093.1 ± 6585.9
IFN-γ	ND	68.2 ± 26.6	368.4 ± 108.4*	27.9 ± 26.1^&^
TNF-α	1.0 ± 0.9	203.0 ± 83.9	532.3 ± 157.6	143.9 ± 56.1
RANTES	2972.5 ± 949.8	3041 ± 309.8	4332.2 ± 525.8	1855.9 ± 137.6^&^
MIP-1α	30.3 ± 16.7	83.9 ± 16.1	217.7 ± 59.6	90.4 ± 25.3
MIP-1β	565.7 ± 335.2	983.1 ± 379.5	2932.9 ± 936.0	723.5 ± 230.5
MCP-1	14671.5 ± 4885.0	10083.3 ± 1543.5	24807.3 ± 7309.5	13074.2 ± 1054.6

Serum cytokine and chemokine concentrations were analyzed with the Bio-Plex Pro^™^ Mouse Cytokine Panel Plex (Bio-Rad). *p < 0.05 vs. the control, ^&^ *p* < 0.05 vs. the tourniquet only (Two-way ANOVA). *n* = 5 for the control and hemorrhage only groups, *n* = 10 for the tourniquet only group and *n* = 8 for the hemorrhage + tourniquet group. ND, not detectable.

## Discussion

Limb hemorrhage often occurs in both civilian and military trauma patients (Morsey et al., 2003; Arora et al., 2015; Chavez et al., 2016; De Rosa et al., 2018; Leurcharusmee et al., 2018; Kasepalu et al., 2020; BenÍtez et al., 2021; Pottecher et al., 2021; Hinojosa-Laborde et al., 2022; Paquette et al., 2022). Tourniquets are an effective first-line therapy to stop extremity bleeding. In practical terms, there would be no reason to deploy tourniquets in the absence of active hemorrhage. However, conventional murine models in the literature were produced with application of tourniquets without hemorrhage (Klausner et al., 1989; Adachi et al., 2006; Hsu et al., 2012; Yang et al., 2012; Mansour et al., 2014; Kao et al., 2015a; Kao et al., 2015b; Karahan et al., 2016; Onody et al., 2016; Foster et al., 2017; Shih et al., 2018; İnce et al., 2019; Packialakshmi et al., 2022). We describe a new TILLIR injury model, which was produced by first inducing hemorrhage then applying tourniquets.

We found that this new model resulted in a greater decline in renal function and damage (as defined by tGFR, BUN, KIM-1 and urine output) compared to either hemorrhage or tourniquet placement alone. There was also the indication that combined hemorrhage and tourniquet application increased histologic damage to the liver and lung. These data indicate that hemorrhage exacerbates tourniquet-induced organ damage. These findings are consistent with the observations from clinical studies showing that hemorrhage markedly increases AKI incidence in trauma patients. For example, a 3-year multicenter observational study found that the rate of AKI in trauma patients was higher in those that presented with hemorrhagic shock (42%) compared to patients presenting without hemorrhagic shock (13%) (Harrois et al., 2018).

Multiple types of tourniquets including McGivney hemorrhoidal ligator band, controlled tension tourniquet and orthodontic rubber band have been used in murine models (Crawford et al., 2007). The orthodontic rubber band stops blood flow to the hindlimb similarly as other two types of tourniquets in mice, but causes significantly less neuromuscular dysfunction (Crawford et al., 2007). Therefore, orthodontic rubber bands have become the method of choice for tourniquets in murine models. Orthodontic rubber bands have been applied to a hindlimb either unilaterally for 3–4 h (Kato et al., 2009; Corrick et al., 2018) or bilaterally for 2–3 h (Kato et al., 2009; Yang et al., 2012). Unilateral application of tourniquets is advantageous to study neuromuscular injury in the hindlimb, because the contralateral hindlimb can serve as a control. Bilateral application of tourniquets induces severe systemic inflammation and is a good model to study tourniquet-induced secondary organ injures. As such, bilateral application of tourniquets has been used to induce acute kidney, lung and liver injures in a majority of studies (Wunder et al., 2005; Yang et al., 2012; Shih et al., 2018; Wang et al., 2022). We bled mice and then applied orthodontic rubber bands bilaterally only for 80 min. It appears that shorter times are required for tourniquets to induce organ damage in the presence of hemorrhage. However, the diameters and force of orthodontic bands used in the previous studies are not available (Wunder et al., 2005; Yang et al., 2012; Shih et al., 2018; Wang et al., 2022), therefore the variations in the severity of ischemia between our and previous studies cannot be ruled out as a potential cause. In the present study we measured GFR with a transdermal method as opposed to the serum creatinine levels, which is less sensitive than the transdermal method (Yang et al., 2012; Scarfe et al., 2018).

It is noteworthy that tourniquet alone for 80 min in the present study only induced mild AKI, whereas tourniquet alone for 76 min induced severe AKI in our previous study (Packialakshmi et al., 2022). The reason is that mice received 1 mL Ringer’s solution in the present study, whereas mice in the previous study did not. It is well known that fluid resuscitation is beneficial to most types of AKI and therefore it is commonly done in the clinical setting (Prowle and Bellomo, 2015; Michelsen et al., 2019). This is another factor that makes our new model of TILLIR more clinically relevant.

Tourniquets alone increased serum levels of various cytokines and chemokines as compared with the control. Yet, addition of hemorrhage had no significant effects on tourniquet-induced cytokines and chemokines except for reducing tourniquet-induced increase in IFN-γ (Table 2). However, whether tourniquets induce increases in cytokine and chemokine levels in the kidney and whether adding hemorrhage enhances the effects of the cytokines and chemokines remain unknown. Furthermore, we measured the serum cytokine and chemokine levels at the end of the experiments. Whether hemorrhage alters tourniquet-induced cytokines and chemokines more acutely remains to be determined.

This model still has several limitations. The amount of hemorrhage was relatively modest (up to 15% of blood volume). The impact of more severe hemorrhage on TILLIR injury remains to be elucidated. Mice were resuscitated with Ringer’s solution instead of blood products, which are the first line resuscitation for hemorrhage clinically. This model lacks a skeletal muscle injury component, which is known to exacerbate AKI in rats (Xiang et al., 2021). Further, only male mice were used in the present study.

In summary, we have developed a clinically relevant mouse model of TILLIR injury by incorporating hemorrhage before applying tourniquets. With this model we have found that hemorrhage exacerbates TILLIR-induced AKI and also potentiated TILLIR-induced structural damages in the lung and liver. Further work with this model can better define the pathophysiology of TILLIR-induced multi-organ injury and identify potential therapeutic targets.

## Data Availability

The original contributions presented in the study are included in the article/supplementary material, further inquiries can be directed to the corresponding author.

## Abbreviations

AKI, acute kidney injury; KIM-1, kidney injury molecule-1; BUN, serum urea nitrogen; tGFR, transcutaneous glomerular filtration rate; TILLIR, tourniquet-induced lower limb ischemia reperfusion.
